# Persistent digestive disorders in the tropics: causative infectious pathogens and reference diagnostic tests

**DOI:** 10.1186/1471-2334-13-37

**Published:** 2013-01-24

**Authors:** Sören L Becker, Jürg Vogt, Stefanie Knopp, Marcus Panning, David C Warhurst, Katja Polman, Hanspeter Marti, Lutz von Müller, Cedric P Yansouni, Jan Jacobs, Emmanuel Bottieau, Moussa Sacko, Suman Rijal, Fransiska Meyanti, Michael A Miles, Marleen Boelaert, Pascal Lutumba, Lisette van Lieshout, Eliézer K N’Goran, François Chappuis, Jürg Utzinger

**Affiliations:** 1 Department of Epidemiology and Public Health, Swiss Tropical and Public Health Institute, Basel, Switzerland; 2 University of Basel, Basel, Switzerland; 3 Institute of Medical Microbiology and Hygiene, University of Saarland Hospital, Homburg/Saar, Germany; 4 Department of Virology, University of Freiburg, Freiburg, Germany; 5 Faculty of Infectious and Tropical Diseases, London School of Hygiene and Tropical Medicine, London, United Kingdom; 6 Department of Biomedical Sciences, Institute of Tropical Medicine, Antwerp, Belgium; 7 Department of Medical Services and Diagnostic, Swiss Tropical and Public Health Institute, Basel, Switzerland; 8 J.D. MacLean Centre for Tropical Diseases, and Divisions of Infectious Diseases and Medical Microbiology, McGill University Health Centre, Montreal, Canada; 9 Department of Clinical Sciences, Institute of Tropical Medicine, Antwerp, Belgium; 10 Institut National de Recherche en Santé Publique, , Bamako, Mali; 11 Department of Internal Medicine, B P Koirala Institute of Health Sciences, Dharan, Nepal; 12 Centre for Tropical Medicine, Faculty of Medicine, Gadjah Mada University, Yogyakarta, Indonesia; 13 Department of Public Health, Institute of Tropical Medicine, Antwerp, Belgium; 14 Institut National de Recherche Biomédicale, Kinshasa, Democratic Republic of the Congo; 15 Université de Kinshasa, Kinshasa, Democratic Republic of the Congo; 16 Department of Parasitology, Leiden University Medical Center, Leiden, The Netherlands; 17 Unité de Formation et de Recherche Biosciences, Université Félix Houphouët-Boigny, Abidjan, Côte d’Ivoire; 18 Département Environnement et Santé, Centre Suisse de Recherches Scientifiques en Côte d’Ivoire, Abidjan, Côte d’Ivoire; 19 Division of Humanitarian and International Medicine, Geneva University Hospitals, Geneva, Switzerland

**Keywords:** Bacteria, Clinical microbiology, Diagnosis, Digestive disorders, Gastroenterology, Helminths, Intestinal protozoa, Persistent diarrhoea, Virus

## Abstract

**Background:**

Persistent digestive disorders account for considerable disease burden in the tropics. Despite advances in understanding acute gastrointestinal infections, important issues concerning epidemiology, diagnosis, treatment and control of most persistent digestive symptomatologies remain to be elucidated. Helminths and intestinal protozoa are considered to play major roles, but the full extent of the aetiologic spectrum is still unclear. We provide an overview of pathogens causing digestive disorders in the tropics and evaluate available reference tests.

**Methods:**

We searched the literature to identify pathogens that might give rise to persistent diarrhoea, chronic abdominal pain and/or blood in the stool. We reviewed existing laboratory diagnostic methods for each pathogen and stratified them by (i) microscopy; (ii) culture techniques; (iii) immunological tests; and (iv) molecular methods. Pathogen-specific reference tests providing highest diagnostic accuracy are described in greater detail.

**Results:**

Over 30 pathogens may cause persistent digestive disorders. Bacteria, viruses and parasites are important aetiologic agents of acute and long-lasting symptomatologies. An integrated approach, consisting of stool culture, microscopy and/or specific immunological techniques for toxin, antigen and antibody detection, is required for accurate diagnosis of bacteria and parasites. Molecular techniques are essential for sensitive diagnosis of many viruses, bacteria and intestinal protozoa, and are increasingly utilised as adjuncts for helminth identification.

**Conclusions:**

Diagnosis of the broad spectrum of intestinal pathogens is often cumbersome. There is a need for rapid diagnostic tests that are simple and affordable for resource-constrained settings, so that the management of patients suffering from persistent digestive disorders can be improved.

## Background

Diarrhoeal diseases and other digestive disorders are leading causes of morbidity and mortality worldwide, with the highest burden concentrated in tropical and subtropical areas that often lack access to clean water and adequate sanitation, and where hygienic conditions are generally poor [1]. According to the World Health Organization (WHO), diarrhoea is classified into three different categories, namely (i) acute watery diarrhoea (lasting several hours or days); (ii) acute bloody diarrhoea (synonymous: dysentery); and (iii) persistent diarrhoea (lasting 14 days or longer) [2]. ‘Chronic diarrhoea’ is often referred to as an individual term applicable to diarrhoea lasting more than 4–6 weeks, but it still lacks an unambiguous definition.

With an estimated burden of 89.5 million disability-adjusted life years (DALYs) caused in 2010, diarrhoeal diseases rank fourth in the recently published Global Burden of Disease Study [3]. Acute diarrhoeal episodes are mainly due to bacterial and viral pathogens that may cause a variety of clinical syndromes ranging from self-limiting events to life-threatening diseases. Children are most vulnerable and diarrhoeal diseases were responsible for more than 1.4 million deaths in 2010, ranking this disorder at position seven on the main causes of death [4]. In the last decades, concerted efforts have considerably improved our understanding of the epidemiology, diagnosis, treatment and control of many diarrhoeagenic pathogens globally, for instance due to the introduction of rotavirus vaccination programmes in many countries since 2006 [5]. As a result, mortality due to diarrhoeal diseases has been reduced from an estimated 2.5 million in 1990 to just under 1.5 million in 2010, a decrease of 42% [4]. However, few research activities have focused on the investigation of persistent diarrhoea and non-acute abdominal pain due to digestive disorders in the tropics. Hence, little is known regarding its aetiology, epidemiology and disease burden. It is widely acknowledged that intestinal parasites, particularly helminths and intestinal protozoa play a major role as causative agents of persistent digestive symptomatologies [6].

Infections with helminths and intestinal protozoa belong to the neglected tropical diseases, along with other diseases caused by bacterial (e.g. Buruli ulcer), viral (e.g. dengue) and fungal infections (e.g. mycetoma) [7]. More than 5 billion people are at risk of neglected tropical diseases, with the common soil-transmitted helminths (i.e. *Ascaris lumbricoides*, hookworm and *Trichuris trichiura*), exhibiting the widest geographical distribution [8]. Due to their intimate connection with poverty, the highest prevalences of neglected tropical diseases are observed in remote rural and deprived urban settings in the developing world [7,9,10]. Neglected tropical diseases drain the social and economic development in endemic countries and they negatively impact on people’s quality of life and well-being at all levels [11-15].

A major challenge in the clinical management of persistent digestive disorders is the weakness of health systems in many low-income countries [16-18]. Hence, affected people might only seek care at a late stage in their therapeutic itinerary, usually at primary health care centres [19,20]. However, these primary health care centres are often under-staffed and ill-equipped, resulting in a low quality of care. The causes of persistent diarrhoea and other digestive disorders are frequently misdiagnosed due to the often unspecific clinical presentations and the absence of evidence-based algorithms for in-depth investigation [7,21]. The notorious underfinancing of health systems in many tropical and subtropical countries explains the severe neglect of laboratory networks and the only limited availability of basic tests for diagnostic services (e.g. direct faecal smears for helminth diagnosis or blood films for malaria diagnosis) [22]. Hence, in many developing countries, current diagnostic and treatment algorithms are often empirical, whereas local prevalence data and differential diagnoses are rarely taken into account at the primary care level.

Against this background, NIDIAG, an international collaboration on integrated diagnosis-treatment platforms, funded by the European Commission, sets out to develop an improved system for delivering primary health care in resource-constrained settings and proposes an integrated approach to this challenge. Emphasis is placed on a patient-centred approach starting from the presentation at the primary health care level of a clinical syndrome that might be due to ‘common’ pathogens. Three clinical syndromes will be investigated in the NIDIAG framework, namely (1) neurological disorders [23]; (2) persistent fever [24]; and (3) digestive disorders. Here, we focus on digestive disorders, which we define as (i) persistent (≥2 weeks) abdominal pain; (ii) persistent (≥2 weeks) diarrhoea (dysenteric or non-dysenteric); and/or (iii) blood in the stool. These digestive disorders will be investigated at different study sites in tropical areas of Africa (Côte d’Ivoire and Mali) and Asia (Indonesia and Nepal). Before clinically applicable diagnosis-treatment algorithms can be developed, the following major challenges/open issues have to be addressed. Firstly, few studies analysed the spectrum of intestinal pathogens causing persistent digestive disorders in the tropics. Therefore, epidemiological investigations targeting all potential pathogens are desirable to define the most common bacteria, parasites and viruses in the different study settings. Secondly, most diagnostic tests have only been validated in Western settings, and hence their diagnostic accuracy in the tropics remains to be determined.

In this manuscript, pursuing an extensive literature review complemented with expert opinion, we provide an overview of potential pathogens (bacterial, parasitic and viral) that might give rise to digestive disorders as defined above. Available diagnostic tests for the identified pathogens are summarised and reviewed, and we propose pathogen-specific reference tests to be utilised for an in-depth diagnostic work-up of symptomatic patients in the different study sites.

## Methods

### Framework

A symptomatology according to the aforementioned inclusion criteria for the syndrome of digestive disorders is likely to be caused by a large variety of infections, but also non-infectious diseases. For example, blood in the stool, accompanied by persistent abdominal pain, may be indicative of colorectal carcinoma or inflammatory disorders (e.g. Crohn’s disease and ulcerative colitis), but may also be a sign of *Schistosoma mansoni* (a helminth) or *Entamoeba histolytica* (an intestinal protozoon) infection. The aim of the NIDIAG project is to develop evidence-based diagnosis-treatment algorithms that can easily be applied in resource-constrained health care settings. As neither diagnosis nor treatment of many non-infectious diseases are currently feasible in remote rural areas of most developing countries, only infectious aetiologies of digestive disorders that may cause severe disease and that are treatable will be thoroughly assessed within the frame of the NIDIAG project and were therefore prioritised in our literature search.

### Search strategy, data extraction and analysis

We performed a literature review to identify and define the bacterial, parasitic and viral pathogens that may give rise to persistent diarrhoea and chronic digestive disorders, and to obtain information on their respective diagnostic methods in order to describe appropriate reference laboratory tests. Since the role of fungi as causative pathogens of gastrointestinal infections is still under debate, fungal infections were not included in this review [25]. The available literature was reviewed by three independent groups. The results were compared, discussed and finally synthesised. Additionally, a number of experts were consulted to complement the literature review.

In a first step, we examined a series of textbooks pertaining to medical bacteriology, parasitology and virology. Moreover, we searched the electronic database MEDLINE/PubMed for infectious pathogens that may cause digestive disorders as defined in the inclusion criteria. After having identified a set of more than 30 pathogens, we searched the database with the following search term for all infectious agents: “disease name/[Mesh]” and the subheading “diagnosis” (e.g. “ascariasis/diagnosis” [Mesh]). The focus of the MEDLINE/PubMed search was on established laboratory tests as well as on newer diagnostic methods, which have been validated recently or are currently under validation (e.g. studies objectively assessing the diagnostic accuracy of different tests). Hence, we primarily focused on reviews, comparative studies and evaluation studies. Our search had no language or other restrictions and we included studies that were published until mid-April 2012.

## Results

Our literature review revealed more than 30 bacterial, parasitic and viral pathogens that may cause persistent digestive disorders. Many of these infectious agents are epidemiologically well characterised in Western settings, while data regarding their occurrence in tropical and subtropical areas are scarce and often contradictory [6,26-28]. Table 1 provides a list of all selected pathogens and typical clinical characteristics that may assist clinicians to curtail their differential diagnosis. However, pathogen-specific diagnosis can rarely be done based on the clinical presentations, and hence additional diagnostic tools are needed.

**Table 1:** Overview of intestinal pathogens (bacteria, intestinal protozoa, helminths and viruses) that may cause persistent digestive disorders in infected individuals

**Enteric pathogen**	**Persistent diarrhoea**	**Persistent abdominal pain**	**Blood in the stool**
**Bacteria**			
*Aeromonas* spp.	+	–	–
*Campylobacter jejuni*, *C. coli*	+	+	+
*Clostridium difficile*	+	+	+
*Escherichia coli*			
Enteroaggregative *E. coli* (EAEC)	+	+	+
Enteropathogenic *E. coli* (EPEC)	+	+	–
Enteroinvasive *E. coli* (EIEC)	+	+	+
Enterohaemorrhagic *E. coli* (STEC/EHEC)	+	+	+
Enterotoxigenic *E. coli* (ETEC)	+	+	–
Diffusely adherent *E. coli* (DAEC)	+	–	–
*Mycobacterium tuberculosis* and atypical mycobacteria	+	+	+
*Plesiomonas shigelloides*	+	–	–
*Salmonella enterica* (typhoidal and non-typhoidal serovars)	+	+	+
*Shigella* spp.	+	+	+
*Tropheryma whipplei*	+	–	–
*Vibrio* spp.	+	–	–
*Yersinia enterocolitica*, *Y. pseudotuberculosis*	+	–	–
**Intestinal protozoa**			
*Balantidium coli*	+	+	+
*Blastocystis hominis*^a^	(+)	(+)	–
*Cryptosporidium* spp.	+	+	–
*Cyclospora cayetanensis*	+	–	–
*Dientamoeba fragilis*^a^	+	+	–
*Entamoeba histolytica*	+	+	+
*Giardia intestinalis* (syn.: *G. lamblia* and *G. duodenalis*)	+	+	–
*Isospora belli* (syn.: *Cystoisospora belli*)	+	(+)	–
Species of microsporidia	+	+	–
**Helminths**			
Cestodes			
* Diphyllobothrium latum*	+	+	–
* Hymenolepis* spp.	+	–	–
* Taenia* spp.	+	+	–
Nematodes			
* Ascaris lumbricoides*	+	+	–
* Capillaria philippinensis*	+	+	–
Hookworm (*Ancylostoma duodenale* and *Necator americanus*)	+	+	–
* Strongyloides stercoralis*	+	+	(+)
* Trichuris trichiura*	+	+	–
Trematodes			
Intestinal flukes	+	+	–
Intestinal blood flukes: *Schistosoma mansoni*, *S. intercalatum*, *S. japonicum*, *S. mekongi*	+	+	+
**Viruses**			
Adenovirus	+	–	–
Astrovirus	(+)	–	–
Bocavirus	(+)	–	–
Coronavirus	(+)	–	–
Cytomegalovirus (CMV)	+	+	+
Enterovirus	+	–	–
Human immunodeficiency virus (HIV-1/2)	+	+	–
Norovirus	+	–	–
Parechovirus	(+)	–	–
Rotavirus	+	+	–
Sapovirus	(+)	–	–

+, existing risk; (+), low risk; –, no risk.^a^ There is an ongoing debate whether these intestinal protozoa have pathogenic potential or should rather be seen as simple commensals of the gastrointestinal tract [29,30].

The large number of available diagnostic tests for the selected pathogens is a challenge for providing the single most accurate method for a given pathogen. Hence, we classified the different methods into four diagnostic categories, namely (i) microscopy; (ii) culture; (iii) immunology (including enzyme immunoassays (EIA), serotyping of isolates and serology); and (iv) molecular biological diagnosis (e.g. polymerase chain reaction (PCR) assays and DNA sequencing). Selection of a reference test for each specific pathogen is primarily based on the sensitivity and specificity of the test as well as practical considerations (e.g. costs, ease of application, availability, etc.). The results are presented in Table 2 (bacteria), Table 3 (intestinal protozoa), Table 4 (helminths) and Table 5 (viruses). Specific issues on the diagnostic work-up are summarised in the following sub-chapters.

**Table 2:** Diagnostic tests for important bacterial pathogens that may cause persistent digestive disorders

**Infectious pathogen**	**Diagnostic method**				
	**Microscopy**	**Stool culture**	**Immunology**	**Molecular biology (PCR)**	**Reference(s)**
*Aeromonas* spp.	-^a^	**Culture on cefsulodin-irgasan-novobiocin (CIN) or selective*Aeromonas*agar**	-	(Experimental, not validated)	[31]
*Campylobacter jejuni*, *C. coli*	Darkfield microscopy: motile, curved or S-shaped rods (suggestive of *Campylobacter* spp.)	**Culture on selective medium**^**b**^**(42°C, microaerophilic conditions)**	· **Faecal antigen enzyme immunoassay:*Campylobacter*-specific antigen (SA)**	*hipO* gene (*C. jejuni*), *glyA* gene (*C. coli*)	[32]
			· Serology (important for diagnosis of postinfectious immunological diseases)		
*Clostridium difficile*	-^c^	**Culture on selective medium, e.g. cycloserin-cefoxitin-fructose agar (CCF) + toxigenic culture**	· **2-step algorithm:**	Toxin genes (increasingly being used in clinical routine)	[33-35]
			**1) Screening: EIA for glutamate dehydrogenase (GDH)**		
			**2) ELISA for detection of toxin A and B**		
			· Cell cytotoxicity assay for detection of toxin A and B		
*Escherichia coli*					
Enteroaggregative	-^a^	**HEp-2 cell adherence assay (following incubation in Luria broth)**	· Serology: antibody response against Plasmid-encoded toxin (*Pet*)	*AggR, CVD432, EAST1* (most common virulence factors, not always present)	[36]
*E. coli* (EAEC)			· ELISA: secretory immunoglobulin A response to EAEC		
Enteropathogenic *E. coli* (EPEC)	-^a^	Culture on MacConkey (MAC) agar	-	***eae*gene**	[37]
Enteroinvasive *E. coli* (EIEC)	-^a^	Culture on MAC agar	ELISA: detection of the *ipaC* gene	***ipaH*,*ipaB*genes**	[38]
Enterohaemorrhagic	-^a^	Culture on sorbitol-MAC agar (most O157:H7 strains form sorbitol-negative colonies)	· O157 latex agglutination test	**STEC:*stx1*,*stx2*genes**	[39,40]
*E. coli* (EHEC including STEC)			· **Shiga toxins 1 & 2 (ELISA)**	**EHEC:*stx1/stx2*+ eae gene**	
Enterotoxigenic *E. coli* (ETEC)	-^a^	Culture on MAC agar	Several immunoassays for toxin detection	***stla/stlb*and*lt*genes**	[41]
Diffusely adherent *E. coli* (DAEC)	-^a^	**HEp-2 cell adherence assay (following incubation in Luria broth)**	-	*daaD* gene	[42]
*Mycobacterium tuberculosis* and atypical mycobacteria	- **Histopathological examination of intestinal biopsies**	**Culture of biopsy material**	· Interferon-gamma-release assay (IGRA) on heparinised blood samples	Nucleic acid amplification tests (lacks sensitivity for diagnosis of extrapulmonary tuberculosis)	[43,44]
	- Acid-fast stain (e.g. Ziehl-Neelsen, Kinyoun, Auramin)		· Tuberculin skin test		
*Plesiomonas shigelloides*	-^a^	**Culture on CIN agar**	-	-	
*Salmonella enterica* (typhoidal and non- typhoidal serovars)	-^a^	· **Culture**^**d**^**from blood and/or bone marrow** (enteric fever)	· Serotyping of isolates (Vi antigen)	(Mainly for research purpose)	[45-47]
		· Culture^d^ from stool or duodenal aspirate (typhoidal and non-typhoidal salmonellosis)	· ELISA: detection of *S. typhi* antigens (blood)		
			· Widal agglutination test (commonly used in Africa)		
*Shigella dysenteriae*, *S. flexneri*, *S. boydii*, *S. sonnei*	-^a^	**Culture on MAC, XLD, HE or Leifson agar**	**Agglutination tests to detect serogroup and serotype**	*ipaH*, *ipl* genes	[48]
*Tropheryma whipplei*	**Histopathological examination of PAS-stained intestinal biopsies: sickleform particle-containing cells**	(Only in highly specialised laboratories)	Immunohistochemistry on PAS-positive biopsy material	***whip1, whip2*genes**	[49]
*Vibrio* spp.	Darkfield microscopy: comma-shaped, motile bacteria (highly suggestive of *Vibrio* spp.)	**Culture on TCBS agar**	-	PCR for species differentiation (*V. cholerae, V. parahaemolyticus, V. vulnificus*)	[50,51]
*Yersinia enterocolitica*, *Y. pseudotuberculosis*	-^a^	**Culture on CIN agar**	Serology (important for diagnosis of postinfectious immunological diseases)	PCR (reference laboratories and research purposes)	[52]

The laboratory techniques are divided into different categories and recommended tests for each pathogen are highlighted.

^a^ Gram staining of stool samples can be useful to evaluate the presence of leucocytes, but is not helpful to differentiate between pathogenic bacteria and apathogenic microbial flora.

^b^ Commonly employed selective media for detection of *Campylobacter* spp. include charcoal-cefoperazone-deoxycholate agar, *Campylobacter* blood agar plate, and cefoperazone-vancomycin-amphotericin agar [53].

^c^ Detection of *C. difficile* in the Gram stain is not adequate to differentiate between clinical infection and simple colonisation with *C. difficile*[54].

^d^ Commonly employed selective media for growth of *S. enterica* are MAC, XLD, HE, Leifson agar or other chromogenic media.

**Table 3:** Diagnostic tests for important intestinal protozoa that may cause persistent digestive disorders

**Infectious pathogen**	**Diagnostic method**				
	**Microscopy**	**Stool culture**	**Immunology**	**Molecular biology (PCR)**	**Reference(s)**
*Balantidium coli*	**Stool microscopy**	-	-	-	[55]
	· **Wet mount smears (unstained or iodine stain)**				
	· **Concentration techniques (e.g. formalin-ether)**				
	· **Permanent stains (e.g. with iron hematoxylin)**				
*Blastocystis hominis*	**Stool microscopy**	Stool culture on selective liquid media (no routine procedure, but beneficial in microscopically uncertain cases)	(No routine procedure)	(PCR mainly applied in research settings)	[56-58]
	· **Wet mount smears (unstained or iodine stain)**				
	· **Permanent stains (e.g. with trichrome, iron hematoxylin, Giemsa)**				
*Cryptosporidium* spp.	**Stool microscopy**	(No routine procedure)	· **ELISA: faecal antigen detection**	**PCR** (in reference laboratories and for species differentiation)	[59-62]
	· Wet mount smears (unstained or iodine stain)		· Fluorescence microscopy		
	· **Various staining techniques, especially acid-fast stains (e.g. Kinyoun, modified Ziehl-Neelsen)**				
*Cyclospora cayetanensis*	**Stool microscopy**	(No routine procedure)	-	**PCR** (in reference laboratories)	[63]
	· **Wet mount smears (light or epifluorescence microscopy)**				
	· **Concentration techniques (e.g. formalin-ether)**				
	· Acid-fast stains (oocysts are variably acid-fast)				
*Dientamoeba fragilis*	**Stool microscopy on stained smears (e.g. iron-hematoxylin, chlorazol black dye stain)**	(No routine procedure)	**-**	**PCR** (in reference laboratories) on unpreserved stool samples	[64,65]
*Entamoeba histolytica*	**Stool microscopy**	(No routine procedure)	· **ELISA: faecal antigen detection able to distinguish between*E. histolytica*and*E. dispar/moshkovskii*(stool)**	**PCR** (in reference laboratories)	[66-70]
	· **Wet mount smears (trophozoites)**		· Serological antibody detection tests (blood samples)		
	· **Formalin-ether concentration (cysts)**				
	· **Permanent stains**				
*Giardia intestinalis*	**Stool microscopy**	(No routine procedure)	· **ELISA: faecal antigen detection**	**PCR** (in reference laboratories)	[60,71]
(syn.: *G. lamblia* and *G. duodenalis*)	· **Wet mount smears (trophozoites)**				
	· **Formalin-ether concentration (cysts)**				
	· **Permanent stains**				
*Isospora belli* (syn.: *Cystoisospora belli*)	**Stool microscopy**	-	-	**PCR** (in reference laboratories)	[60,72,73]
	· Wet mount smears				
	· Concentration techniques (e.g. formalin-ether)				
	· **Acid-fast stains**				
Species of microsporidia (*Enterocytozoon bieneusi, Encephalitozoon* spp.)	· **Transmission electron microscopy** (gold standard, but not feasible as routine test)	-	Serology: anti-microsporidial antibodies (indirect immunofluorescence assay)	**PCR** (in reference laboratories)	[60,74-76]
	**- Light microscopy** (e.g. Uvitex B, Chromotrope R or Calcofluor White stain)				

The laboratory techniques are divided into different categories and recommended tests for each pathogen are highlighted.

**Table 4:** Diagnostic tests for important helminths that may cause persistent digestive disorders

**Infectious pathogen**	**Diagnostic method**				
	**Microscopy**	**Stool culture**	**Immunology**	**Molecular biology (PCR)**	**Reference(s)**
Cestodes					
*Diphyllobothrium latum*	**Stool microscopy: identification of eggs or proglottids**	-	-	PCR and sequencing for species differentiation (for epidemiological purpose)	[77,78]
	· **Wet preparation**				
	· **Ethyl-acetate or formalin-ether-based concentration techniques**				
	· **Sedimentation techniques**				
*Hymenolepis* spp.	**Stool microscopy**	-	-	PCR in research settings (for epidemiological purpose)	[79]
	· **Kato-Katz method**				
	· **Ethyl-acetate or formalin-ether-based concentration techniques**				
	· **Sedimentation techniques**				
	· **FLOTAC**				
*Taenia* spp.	**Stool microscopy**	-	· Coproantigen EIA	PCR for species differentiation	[80]
	· Perianal egg detection		· Serology: detection of specific circulating antibodies against *T. solium*		
	· (Graham’s test applying adhesive tape)				
	· Examination of tapeworms from purges				
Nematodes					
*Ascaris lumbricoides*	**Stool microscopy: egg detection**	-	-	PCR in research settings (for epidemiological purpose)	[81-83]
	· **Kato-Katz method**				
	· **Ethyl-acetate or formalin-ether-based concentration techniques**				
	· **Sedimentation techniques**				
	· **FLOTAC**				
*Capillaria philippinensis*	**Stool microscopy: egg detection**	-	-	-	[84,85]
	· **Ethyl-acetate or formalin-ether-based concentration techniques**				
	· **Sedimentation techniques**				
	· (Kato-Katz method: great care is indicated to distinguish between *T. trichiura* and *C. philippinensis* eggs)				
Hookworms (*Ancylostoma duodenale*, *Necator americanus*)	**Stool microscopy: egg detection**	Culture on Koga agar and subsequent microscopic identification of larvae		PCR mainly applied in research settings (for epidemiological purpose)	[81-83]
	· **Kato-Katz method**				
	· **Ethyl-acetate or formalin-ether-based concentration techniques**				
	· **Sedimentation techniques**				
	· **FLOTAC**				
*Strongyloides stercoralis*	· **Stool: microscopy following Baermann funnel concentration**	**Culture on Koga agar and subsequent microscopic identification of larvae**	· ELISA tests detecting serum antibodies or faecal antigens	PCR applied in research settings (for epidemiological purpose) and increasingly used for individual patient management	[86,87]
	· Microscopy of sputum, bronchoalveolar lavage, duodenal aspirate, skin biopsy		· Indirect fluorescent antibody test		
*Trichuris trichiura*	**Stool microscopy: egg detection**	-	-	-	[81,82]
	· **Kato-Katz method**				
	· **Ethyl-acetate or formalin-ether-based concentration techniques**				
	· **Sedimentation techniques**				
	· **FLOTAC**				
Trematodes					
Intestinal flukes	**Stool microscopy: egg detection**	-	ELISA to detect worm-specific antibodies or antigens in serum or stool	PCR applied in research settings (for epidemiological purpose)	[88]
	· **Kato-Katz method**				
	· **Ethyl-acetate or formalin-ether-based concentration techniques**				
	· **Stoll’s dilution**				
	· **Sedimentation techniques**				
	· **FLOTAC**				
Intestinal blood flukes: *Schistosoma mansoni*, *S. intercalatum*, *S. japonicum*, *S. mekongi*	**Stool microscopy: egg detection**	-	- ELISA to detect serum antibodies or worm-specific antigens in serum or urine	PCR applied in research settings for epidemiological purpose and increasingly used for individual patient management	[89]
	· **Kato-Katz method**		- RDT to detect CCA or CAA antigen in serum or urine (for *S. mansoni*)		
	· **Ethyl-acetate or formalin-ether-based concentration techniques**				
	· **Stoll’s dilution**				
	· **Sedimentation techniques**				
	· **FLOTAC** (first experiences for *S. mansoni*)				
	Miracidium-hatching test from stool samples				

The laboratory techniques are divided into different categories and recommended tests for each pathogen are highlighted.

**Table 5:** Diagnostic tests for important viral pathogens that may cause persistent digestive disorders

**Infectious pathogen**	**Diagnostic method**				
	**Electron microscopy**	**Cell culture**	**Immunology**	**Molecular biology (PCR)**	**Reference(s)**
**Viruses**					
Adenovirus	Low sensitivity (>10^6^ viral particles/ml)	A549-, HEp-2-, HEK-cells	Antigen detection in faecal samples (ELISA, immunochromatography)	**PCR**	[90]
Astrovirus	Low sensitivity (>10^6^ viral particles/ml)	CaCO-2-, LLC-MK2-cells	Antigen detection (ELISA)	**RT-PCR**	[91]
Bocavirus	**-**	**-**	**-**	**PCR**	[92]
Coronavirus	**-**	**-**	**-**	**RT-PCR**	[93]
Cytomegalovirus (CMV)	**-**	HFF-, MRC-5 cells	· pp65 antigen detection (immunofluorescence)	**PCR**	[91]
		CMV-immediate early1-pp72-antigen in HFF	· (CMV-specific antibody seroconversion)		
Enterovirus	**-**	MRC-5-, HEp-2-, Vero-cells	**-**	**RT-PCR**	[94]
Human immunodeficiency virus (HIV-1/2)	**-**	HUT-78-, CEM-MOLT4-cells	· **Immunoassay (e.g. 4th generation)**	**RT-PCR**	[95,96]
			· **Western Blot**		
Norovirus	Sensitivity 10^5^-10^6^ viral particles/ml	**-**	Antigen detection faecal samples (EIA)	**RT-PCR**	[91]
Parechovirus	**-**	**-**	**-**	**RT-PCR**	[97]
Rotavirus	Low sensitivity (>10^6^ viral particles/ml)	MA104-, CaCO-2-cells	Antigen detection in faecal samples (ELISA), rapid tests (ELISA, immunochromatography)	**RT-PCR**	[91]
Sapovirus	**-**	**-**	**-**	**RT-PCR**	[91]

The laboratory techniques are divided into different categories and recommended tests for each pathogen are highlighted.

RT-PCR, reverse transcriptase-polymerase chain reaction.

### Bacterial pathogens

#### Aeromonas spp., Campylobacter jejuni, C. coli, Plesiomonas shigelloides, Salmonella enterica (non-typhoidal serovars, e.g. S. enterica ser. Enteritidis, S. enterica ser. Typhimurium), Shigella spp., Vibrio spp., Yersinia enterocolitica, Y. pseudotuberculosis

A stool culture on selective media is the test of choice to detect these diarrhoeagenic bacteria [31,48,50,52,53]. Different solid media (e.g. selective agar plates containing antibiotics and substances favouring the growth of the sought microorganism) are inoculated with a stool specimen to detect and isolate enteric pathogens. The additional use of a selective enrichment broth is helpful to identify pathogenic bacteria if their presence is quantitatively so low that they might otherwise be overlooked on solid media due to the overgrowth of non-pathogenic intestinal flora. The inoculated media are usually incubated for 24–72 hours at 35°C at ambient atmosphere to allow the bacteria to form macroscopically visible colonies. Of note, *Campylobacter* spp. are isolated using different growth conditions, i.e. incubation at a higher temperature of 42°C in microaerophilic atmosphere [53].

Following the incubation period, the agar plates are examined and morphologically suspicious colonies are identified using different biochemical identification panels or automated phenotypic identification systems (e.g. Vitek®; bioMérieux, Marcy l’Étoile, France). Recently, more rapid identification algorithms making use of mass spectrometry (MS) have successfully been implemented into clinical microbiology laboratories (e.g. matrix-assisted laser desorption ionization time-of-flight (MALDI-TOF) MS (e.g. MicroFlex LT; Bruker Daltonics, Bremen, Germany) [98].

Stool culture remains the diagnostic ‘gold’ standard for enteric pathogenic bacteria disposing certain characteristics which enable them to be selected out of the normal gastrointestinal flora, while other bacteria without such characteristics cannot be distinguished from apathogenic gut bacteria by culture methods alone (see below). Stool culture has important advantages, such as testing of isolated pathogens for antimicrobial susceptibility. As the successful antibiotic treatment of many bacterial intestinal infections requires knowledge of local resistance patterns (e.g. extent of fluoroquinolone-resistant *Campylobacter* strains), stool culture techniques remain mandatory to guide therapeutic interventions. However, these tests are laborious and require experienced personnel, and typically take 48–72 hours to obtain first results. Hence, other, more rapid diagnostic tests (RDTs) have been developed for some pathogens. For *Campylobacter* spp., for example, EIAs detecting a specific antigen in the stool proved to be a sensitive alternative to stool culture with results available within a few hours [32,99]. However, there is no international consensus on immunological assays for detection of *Campylobacter* spp. and no globally validated and standardised approach, so that these tools should not replace the selective stool culture [100]. PCR assays, characterised by high sensitivity and specificity, have been developed for most of the aforementioned bacteria. Thus far, however, integration into clinical routine testing is still limited. Important drawbacks are the high cost, the need for sophisticated laboratory equipment and well-trained laboratory technicians. Moreover, PCR cannot distinguish between dead or alive bacteria and does not allow testing for antimicrobial susceptibility. However, newly developed multiplex PCR assays are increasingly being evaluated as fast screening tests for early detection of various important enteric pathogens. Besides PCR, novel molecular diagnostics are currently being developed and validated for many bacterial and viral pathogens, e.g. loop-mediated isothermal amplification (LAMP) assays. Results obtained thus far are promising [101,102], but it remains to be elucidated whether such nucleic acid amplification tests can be employed on a larger scale in resource-constrained settings in the tropics.

#### Salmonella enterica ser. Typhi/*Paratyphi*

Diagnosis of enteric fever is challenging and often delayed or not performed due to the unavailability of the most sensitive techniques in areas of high endemicity [103]. In these settings, the Widal test (measuring an increasing *S.* Typhi-specific antibody titer over the course of 10 days in patient serum samples) is often the only available test, despite its poor diagnostic performance. Contrary to non-typhoidal salmonellosis, stool culture is not sufficiently sensitive to diagnose infection due to *S.* Typhi/*S.* Paratyphi. Culturing blood and bone marrow is more sensitive, but bone marrow aspiration is only rarely performed in tropical areas due to a lack of adequately equipped hospitals and laboratories [104]. Blood cultures should be obtained during the first week of disease to achieve adequate sensitivity [45]. Serotyping of isolates (e.g. by agglutination of Vi antigen or rapid detection of various antigens or IgM antibodies by different EIA kits) is helpful for a timely diagnosis, but lacks sensitivity and specificity [46]. PCR assays have been developed for different antigens of invasive *S. enterica* serovars, but still need further development and validation before they can be more widely recommended [105].

#### Clostridium difficile

*C. difficile* can be found as part of the physiological intestinal flora, but toxin-producing strains may cause severe diarrhoea, which is most frequently seen in hospitalised patients who recently received antibiotic treatment [33]. A selective stool culture (toxigenic culture, performed on a selective agar medium or after ethanol shock pretreatment) followed by tests for toxin production remains the diagnostic ‘gold’ standard for *C. difficile*[33] and is particularly useful when the quantity of toxins in stool samples is small [34]. A laborious and technically difficult cell culture cytotoxicity assay is still regarded as an alternative reference standard, but is seldom performed in most microbiological laboratories. More recently developed PCR assays targeting a toxin-encoding gene are currently discussed as an alternative method for early diagnosis of *C. difficile* infection. Such molecular methods allow a more precise characterisation of isolated *C. difficile* strains, e.g. ribotype differentiation [35,106,107]. Sensitivity and specificity of PCR have been reported to vary between 85% and 100% [108]. However, various molecular assays exist which are not yet fully standardised, and the diagnostic performance of commercially available kits may differ considerably from in-house molecular testing methods in use at different laboratories. Of note, PCR can only prove the presence of the toxin-encoding gene, but cannot distinguish between asymptomatic carriage and acute infection.

In clinical practice, an easily applicable two-step approach is recommended for rapid and reasonably sensitive diagnosis of *C. difficile*[109]. Firstly, a screening test for *C. difficile*-associated glutamate dehydrogenase (GDH) should be performed to indicate the bacterium’s presence in a stool sample. If positive, it should be followed by a test for toxin production (e.g. toxin A/B EIA). This procedure does not require an extensively equipped laboratory and generates accurate results within a few hours. However, the sensitivity and specificity of this two-step approach are limited, and hence toxigenic culture and PCR testing should always be performed when there is a high clinical suspicion despite negative test results [110].

#### Pathogenic *Escherichia coli* strains

Diagnosis of pathogenic *E. coli* is challenging, as these bacteria constitute an important part of the physiological intestinal flora and only some strains have diarrhoeagenic potential [41]. There are at least six groups of pathogenic *E. coli* strains, namely (i) diffusely adherent (DAEC); (ii) enteroaggregrative (EAEC); (iii) enterohaemorrhagic (EHEC, including STEC = shiga toxin-producing *E. coli*); (iv) enteroinvasive (EIEC); (v) enteropathogenic (EPEC); and (vi) enterotoxigenic *E. coli* (ETEC). Pathogenic *E. coli* strains that carry simultaneously virulence factors from different pathotypes may cause severe clinical outbreaks. In mid-2011 in Germany, for example, the *E. coli* strain O104:H4 (an EAEC capable of EHEC/STEC-specific shiga toxin production) caused 2,987 cases of acute, often severe gastroenteritis and 855 cases of haemolytic-uraemic syndrome which led to 53 deaths [111].

While diagnostic procedures are poorly standardised for the pathotypes DAEC and EAEC, molecular biological testing has revolutionized the diagnostic algorithms for the other diarrhoeagenic *E. coli*. Modern multiplex PCR assays targeting unique genes of EHEC/STEC, EIEC, EPEC and ETEC allow a rapid molecular characterisation of these pathogenic strains. Hence, multiplex PCR assays have become the test of choice with excellent sensitivity and specificity (>99%) [42]. Indeed, these tests have overcome important drawbacks of the classical stool culture, which often detects only some important strains (e.g. in the case of EHEC the O157:H7 strain on Sorbitol-MacConkey agar), but misses others that lack characteristic biochemical properties [39]. However, the integration of such multiplex PCR assays into routine testing of clinical samples remains restricted to well-equipped laboratories, and hence, these molecular techniques are only rarely available in endemic settings in the tropics.

#### Mycobacterium tuberculosis and atypical mycobacteria (e.g. M. avium)

Gastrointestinal tuberculosis is the sixth most common manifestation of extrapulmonary tuberculosis and causes considerable morbidity, including persistent diarrhoea and abdominal pain [112]. Atypical mycobacteria (synonymous: mycobacteria other than tuberculosis, MOTT), particularly *M. avium*, are an important cause of long-lasting diarrhoea and gastrointestinal complaints in HIV-infected individuals. Accurate diagnosis is difficult and relies on in-depth analysis of intestinal biopsy specimens by histopathological examination, microscopy after acid-fast staining (e.g. Ziehl-Neelsen, Auramin or Kinyoun techniques) and culture on selective media suitable for mycobacteria. Unless performed using oil immersion, histopathology often fails to distinguish between gastrointestinal tuberculosis and other granulomatous disorders, such as Crohn’s disease [113,114]. An important drawback when culturing mycobacteria is their slow growth; it might take up to six weeks until cultures become positive. However, culture is the most sensitive technique and remains the diagnostic ‘gold’ standard [115]. Different molecular biological assays have been developed for various mycobacteria, but lack sensitivity for extrapulmonary tuberculosis and have not yet been validated for gastrointestinal tuberculosis [43].

#### Tropheryma whipplei

Whipple’s disease due to infection with *T. whipplei* is a rare disease characterised by chronic diarrhoea, wasting, abdominal pain, arthralgia and various other symptoms associated with organ involvement (e.g. encephalitis and endocarditis) [49]. The infectious agent was not identified until 1961 and many epidemiological and biological features still need to be elucidated [116]. Only highly specialised laboratories are able to grow *T. whipplei* on human fibroblast cells [117,118]. The development of a PCR assay targeting the genes *whip1* and *whip2* has been a major step forward and is nowadays the test of choice, especially in symptomatic patients without typical histopathological findings in intestinal biopsies (sickleform particle-containing cells on periodic acid-Schiff (PAS-)stained biopsy specimens) [49].

### Parasitic pathogens: intestinal protozoa

#### Balantidium coli, Blastocystis hominis, Cryptosporidium spp., Cyclospora cayetanensis, Dientamoeba fragilis, Entamoeba histolytica, Giardia intestinalis (syn.: G. lamblia and G. duodenalis), Isospora belli (syn.: Cystoisospora belli), species of microsporidia

The three main techniques for the diagnosis of human intestinal protozoan infections include (i) light microscopy; (ii) antigen detection (EIAs); and (iii) PCR assays. Since the first description of parasitic intestinal protozoa in human stools, documented by the Dutch microscopist Antony van Leeuwenhoek in 1681 [119], microscopic detection of protozoan cysts and trophozoites has been the most widely used diagnostic approach. On fresh stool samples, direct microscopy is performed by mixing a small amount of faeces with a physiological 0.9% sodium chloride (NaCl) solution. To increase sensitivity, various stool concentration techniques have been developed, making use of either sedimentation or flotation with a formalin-ether concentration technique being the most widely used method in medical laboratories [120,121]. However, the formalin-ether concentration technique lacks sensitivity for several intestinal protozoan species as well as many helminths (described below), and hence there is a pressing need for new and more sensitive microscopic techniques (e.g. FLOTAC) [122] and non-microscopic diagnostics. Staining techniques can be helpful for microscopic parasite identification and might further improve the diagnostic accuracy. Indeed, some intestinal protozoan species require staining of the stool sample to be identified on microscopic examination. For example, acid-fast stains allow detection of *Cryptosporidium* spp., while species of microsporidia are best seen when using an Uvitex B or Calcofluor White stain. Still, correct identification of intestinal protozoan pathogens is challenging even for experienced laboratory technicians and for some species even impossible (e.g. *E. histolytica* based on cysts morphology). For *Cryptosporidium* spp., *E. histolytica* and *G. intestinalis*, sensitive EIAs detecting species-specific antigens in faecal samples have been developed, some of which are highly sensitive and complement microscopic stool examination in many clinical laboratories [123,124]. Especially for the diagnosis of *E. histolytica*, species differentiation based on alternative procedures is compulsory, since microscopy cannot readily distinguish between *E. histolytica* and the non-pathogenic *E. dispar*[66,125,126]. Of note, not all commercially available EIA antigen detection kits are *E. histolytica*-specific and some lack sensitivity, in particular if faecal samples have been stored for several days [67,127]. Over the past several years, highly sensitive PCR assays have been developed and standardised for many intestinal protozoan species. Many of these assays (e.g. *Entamoeba* spp. differentiation by PCR) are currently being integrated into parasitological reference laboratories as an additional diagnostic tool to prove diagnosis in uncertain clinical cases [59,128,129]. Such molecular biological tools are of enormous importance to improve the correct species identification of many intestinal parasites, which are difficult to diagnose using conventional techniques [60,74].

### Parasitic pathogens: helminths

#### Ascaris lumbricoides, Capillaria philippinensis, Diphyllobothrium spp., Hymenolepis *spp.,* hookworm (Ancylostoma duodenale and Necator americanus), Taenia spp., Trichuris trichiura, intestinal flukes

**Figure 1:**
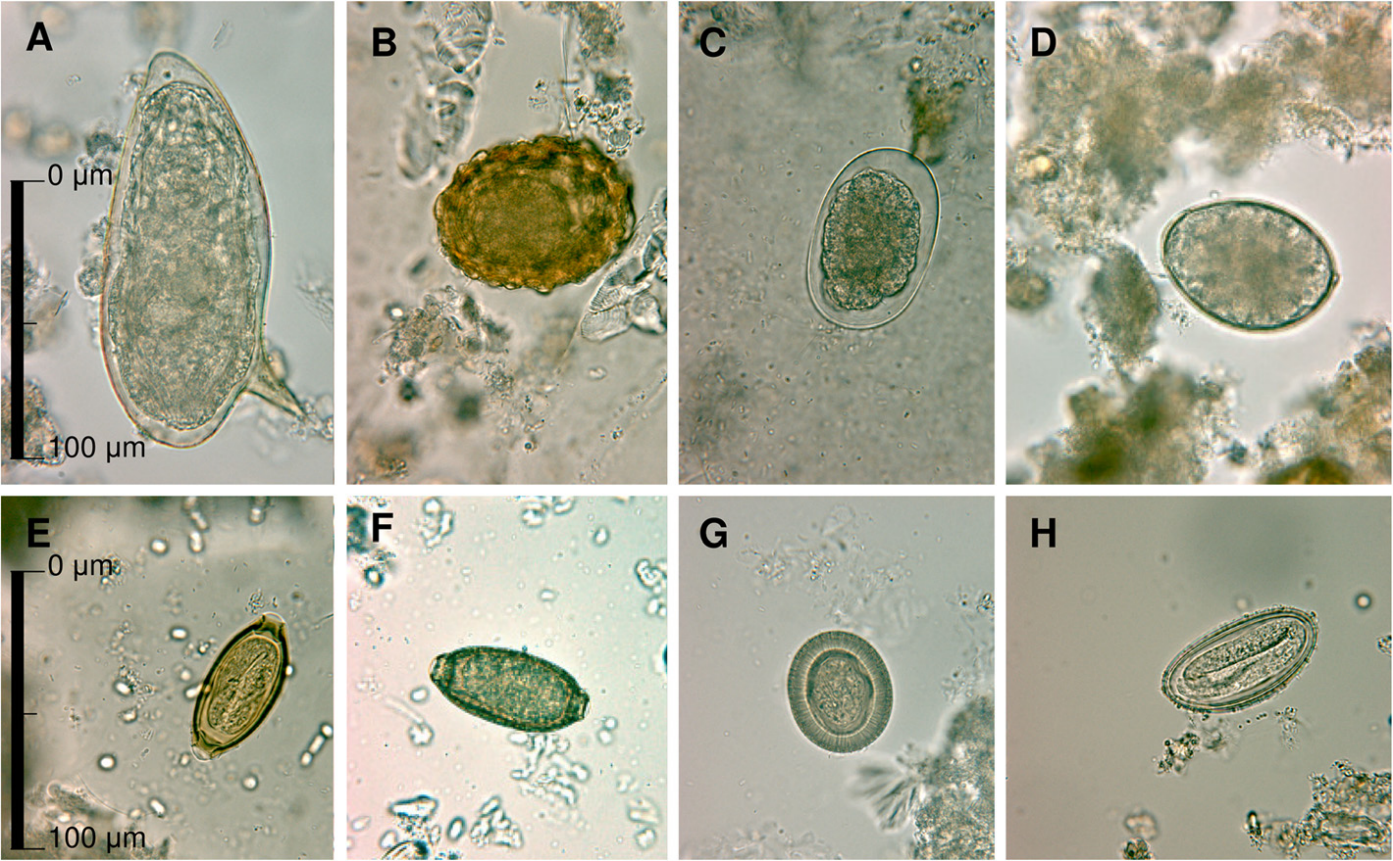
**Morphological features of selected intestinal helminth eggs diagnosed using the formalin-ether concentration technique and standard light microscopy: A, *Schistosoma mansoni*; B, *Ascaris lumbricoides*; C, hookworm; D, *Diphyllobothrium latum*; E, *Trichuris trichiura*; F, *Capillaria *spp.*.*; G, *Taenia *spp.; H, *Enterobius vermicularis.***

Identification of helminth eggs on microscopic stool examination is the reference test for most intestinal helminth species. In hospitals and microbiological laboratories, direct stool examination after prior concentration (e.g. by formalin-ether concentration technique) is most commonly employed, while the Kato-Katz thick smear technique is widely used in epidemiological studies and anthelminthic drug efficacy evaluations in endemic regions [81,130-132]. Direct microscopic examination is a cheap methodology, the microscope slides can rapidly be prepared for examination, and there is no need for sophisticated laboratory equipment. The eggs of most helminth species parasitising humans can easily be distinguished by a trained laboratory technician (see Figure 1 for eight selected helminth eggs). Hence, microscopy remains the standard reference test for *A. lumbricoides*, *T. trichiura*, hookworm, *Capillaria philippinensis*, *Diphyllobothrium* spp., *Hymenolepis* spp., *Taenia* spp. and blood flukes (*Schistosoma* spp.) [82,88,133,134]. However, microscopy is prone to a number of shortcomings. Firstly, microscopy is not very sensitive and especially infections of light intensity can be missed when only a single stool sample is analysed [131,135]. Multiple stool sampling, ideally over several consecutive days, increases the sensitivity [136], as well as the use of different concentration techniques, which are based on sedimentation (e.g. formalin-ether concentration technique), flotation or a combination of both (e.g. McMaster technique and FLOTAC) [135,137-139]. However, these techniques often require access to the power grid, a centrifuge and different chemical reagents, which are not always available in tropical settings. Moreover, the diagnostic sensitivity for different helminth species often varies considerably, and no currently available concentration technique is able to concurrently detect intestinal protozoa and helminths with the same diagnostic accuracy [122,140].

Secondly, microscopy results heavily depend on the quality of the slide preparation and on the experience of the laboratory technician reading the slides. Thirdly, the eggs of some helminth species such as the two hookworm species *A. duodenale* and *N. americanus* are virtually indistinguishable by microscopy. Fourthly, the nematode *Strongyloides stercoralis* can rarely be found when using the aforementioned microscopy techniques, because its larvae already hatch in the intestine and, hence, the eggs are not passed in the faeces [141]. Despite all these constraints, microscopy is an invaluable tool for diagnostic medical parasitology.

New molecular techniques, especially PCR assays, still need to be validated and further developed in different settings. Disadvantages of current PCR tests are their high costs, risk of contamination, the need for high-technology laboratory equipment and constant electric power supply which render their use for routine testing in many developing countries impossible. Indeed, PCR is seldom available in the most affected regions, and its results often do not guide clinicians’ decisions, as empiric treatment with albendazole and mebendazole is commonly employed and effective against many helminth species in endemic areas [7]. Due to the variety of intestinal parasites causing digestive disorders, a multiplex real-time PCR targeting a host of various pathogens is much more desirable than individual PCR assays for each parasite, and such multiplex PCRs have been successfully developed and are increasingly used in reference laboratories in industrialised countries [59,83,142]. However, even these multiplex PCRs can only diagnose a defined host of targeted pathogens, while microscopy may sometimes detect unexpected pathogens that would have been missed by other diagnostic methods.

#### Strongyloides stercoralis

The diagnosis of *S. stercoralis* in human stool samples requires special, often laborious concentration techniques. Most commonly employed are the Baermann funnel and the Koga agar plate [143]. The Baermann method is a concentration technique based on the nematode’s hydrophily and thermophily. It provides results within a few hours and is the technique of choice according to the World Gastroenterology Organization [144], but there is some debate whether it is as sensitive as agar plate cultures [86]. Derived from classical charcoal culture assays and its sequel, the so-called Harada-Mori culture, Koga and colleagues developed a special agar plate to detect *S. stercoralis* and hookworm larvae [145]. The agar plates are stored for 48 hours in a humid chamber and the traces of the helminths can then be seen on the agar and the larvae can easily be collected for microscopic species identification. In contrast to many other helminth infections, where exact species identification often is not necessarily required and clinical symptoms are mild, the recognition of strongyloidiasis and initiation of an effective treatment with ivermectin is essential to prevent potentially life-threatening events due to its ability to cause disseminated hyperinfection in the immunosuppressed population [141,146]. Hence, the aforementioned laborious techniques seem to be justified and a combination of the Baermann funnel and the Koga agar plate method may lead to the most accurate results.

Recently, different PCR assays targeting the helminth’s 18S rRNA [87] or 28S rRNA [147] subunit have been developed. First results are promising, but still need further validation in endemic settings.

#### Schistosoma mansoni, S. mekongi, S. intercalatum and S. japonicum

The microscopic detection of blood fluke eggs in stool specimens still remains the cornerstone of the laboratory diagnosis of intestinal schistosomiasis, as the specificity is high and the costs of equipment are relatively low. However, the sensitivity fluctuates, depending on infection stage and intensity [148]. Hence, concentration methods like an ether-concentration, the Kato-Katz thick smear or the recently developed FLOTAC technique are important tools to increase sensitivity [89]. Moreover, examination of multiple (preferably at least three) stool samples collected on consecutive days is recommended [136,149]. In contrast to other helminth infections, immunological RDTs have been developed for detection of intestinal (*S. mansoni*) and urogenital schistosomiasis (*S. haematobium*). Worm-gut associated glycoproteins, namely circulating cathodic antigen (CCA) and circulating anodic antigen (CAA), can be detected in the serum and the urine of *S. mansoni*-infected individuals using genus-specific monoclonal antibodies [150,151]. Immunochromatographic point-of-care (POC) dipstick or cassette tests for rapid diagnosis of *S. mansoni* via CCA detection in the urine are currently being validated in different epidemiological settings and will potentially become a valuable tool for non-microscopic diagnosis of schistosomiasis in epidemiological studies and clinical practice. Recent studies suggest that the diagnostic accuracy of a single POC-CCA test is considerably more sensitive than a single Kato-Katz thick smear and that a concurrent *S. haematobium* infection does not influence the POC-CCA test results for *S. mansoni* diagnosis, which is an important observation due to the co-endemicity of both blood fluke infections in many tropical areas [152,153]. Hence, antigen RDT assays will likely find their way into clinical practice in the foreseeable future.

PCR assays have been developed and are more sensitive than conventional parasitological and serological methods, but presently, their use is restricted to specialised reference laboratories and research institutions outside endemic areas [154,155].

### Viruses

Viral infections commonly cause acute gastroenteritis with the highest burden concentrated in tropical and subtropical regions of the world. Even though these pathogens mainly lead to short-lasting and self-limiting diarrhoeal diseases, they account for considerable morbidity and even mortality, particularly in children [156]. In general, viral infections rarely cause chronic intestinal diseases, but must not be forgotten as potential pathogens that may give rise to persistent diarrhoea and chronic abdominal pain, particularly in HIV-infected individuals or otherwise immunocompromised hosts.

Traditionally, diagnosis of viral gastroenteritis is based on virus isolation by cell culture, electron microscopy and rapid antigen tests (e.g. latex agglutination or EIAs) [157]. Introduction of molecular methods led to an exponential increase in detection rates and the role of difficult-to-culture pathogens became apparent. From a technical point of view, most rapid tests can be done at the bedside, whereas cell culture, electron microscopy and molecular-based methods require laboratories with sophisticated equipment, experienced staff and appropriate biosafety procedures. This certainly limits the use of the latter methods in resource-constrained settings. Data on sensitivity and specificity of diagnostic tools for virus identification in tropical settings are currently lacking.

#### Adenovirus

Currently, more than 53 types of adenovirus are recognised which can cause a variety of clinical entities, but gastroenteritis is predominantly caused by types 40 and 41 [158,159]. In infected individuals, viral particles are shed in high concentrations. In general, virus isolation followed by serotyping remains the ‘gold’ standard for the detection of all serotypes and is possible on different cell lines (Table 5). Importantly, 293-Graham cells should be used for stool samples as adenovirus species F (adenovirus types 40 and 41) can only be cultivated on this cell line. However, virus isolation is rather laborious and time-consuming in the face of urgent requests for diagnosis. Electron microscopy is possible with high specificity, but low sensitivity. As an alternative method that is particularly useful for examination of stool samples, antigen detection assays using EIA or latex agglutination have been developed [160,161]. These assays are rapid, but displayed varying sensitivities and specificities in studies, and hence should be complemented by alternative methods. Molecular methods, in particular real-time PCR, have demonstrated superior performance over conventional methods and are now the cornerstone for diagnosis in most laboratories, but are seldom available in resource-constrained settings.

#### Astrovirus

Eight serotypes of astrovirus are known. In childhood, astrovirus infection with serotypes 1 and 2 predominate, whereas infection with the other serotypes occurs later in life (>4 years). Prolonged diarrhoea has been associated with astrovirus serotype 3 [162]. In the immunocompetent host, viral shedding occurs for 14–70 hours but may be prolonged in immunosuppressed patients. Virus propagation of astrovirus on CaCO-2 or LLC-MK2 cells remains restricted to expert laboratories and is not recommended for routine diagnostic use. Virus identification by electron microscopy is possible, but appearance of viral particles is not always clear. Recently developed antigen detection kits have proven their suitability and are now widely available for rapid diagnosis. However, sensitivity and specificity of rapid tests in comparison to reverse transcriptase (RT)-PCR have been reported to be comparably low [163]. Real-time RT-PCR is the most sensitive and specific method, but remains restricted to reference laboratories.

#### Bocavirus

Four different species of human bocavirus (hBoV) have been described thus far [164]. The diagnosis of hBoV infection is almost exclusively based on molecular methods. hBoV has not been isolated by cell culture or in an animal model and rapid antigen tests are currently not available. Serology (e.g. using viral-like particles) has been described and can be used to complement diagnosis [165]. A variety of PCR and real-time PCR assays have been described. However, due to prolonged detection of viral DNA at low copy numbers, qualitative detection of hBoV DNA in gastrointestinal samples is not recommended. There are only few data available for hBoV species 2–4 and the relevance as a true human pathogen is still under debate [164].

#### Calicivirus

The family *calicivirus* comprises two human-pathogenic genera, the norovirus and sapovirus [166]. For both genera, virus isolation by cell culture is not possible. Electron microscopy is rather insensitive and rarely detects the viruses if there are fewer than 10^6^ viral particles/ml of stool suspension.

##### Norovirus

Antigen EIAs have been developed and are commercially available for rapid diagnosis. They proved to be a valuable tool especially in outbreaks, but their sensitivity is limited [167]. A recent study from Brazil reported a sensitivity of 87.9% upon use of a 3rd generation norovirus antigen detection kit [168]. More recently, real-time RT-PCR assays have been described and demonstrated excellent sensitivity and specificity [169]. In-house methods as well as commercial kits are widely available and routinely used.

##### Sapovirus

Specific real-time RT-PCR assays have been developed, but there are no comprehensive data evaluating their diagnostic accuracy. However, there are no diagnostic alternatives because rapid antigen tests are not yet available.

#### Coronavirus

Five different human pathogenic coronaviruses are known which can cause respiratory and/or to a lesser extent gastrointestinal symptoms in humans. However, the relevance of coronavirus as a true human enteric pathogen is unclear [93,170]. Conventional virus isolation by cell culture can be done on human embryonal tracheal cells. Electron microscopy is possible for stool samples but displays rather low sensitivity. For coronavirus, antigen tests for stool samples are not available. Molecular methods, e.g. real-time RT-PCR assays are the method of choice for a reliable and rapid diagnosis. However, most in-house methods are restricted to reference laboratories, and hence are not commonly employed around the globe.

#### Cytomegalovirus

In particular immunosuppressed patients are at risk for cytomegalovirus (CMV) infection, which can affect various organ systems, including the gastrointestinal tract [158,171]. Serology represents the method of choice to differentiate primary from secondary infection. Organ-specific diagnosis (e. g. CMV-associated gastrointestinal disease) requires tissue biopsy samples. In combination with histopathology, isolation of CMV by cell culture is recommended. Detection of CMV-DNA by molecular methods alone is not sufficient.

#### Enterovirus

Enteroviruses belong to the family *picornaviridae* and comprise enterovirus group A to D [172]. In general, enteroviruses can cause a broad spectrum of different clinical entities. Gastroenteritis caused by coxsackievirus A is mostly seen in children. Virus isolation is possible on a range of different cell lines (Table 5). Virus typing after isolation is traditionally accomplished by virus neutralisation. Of note, enteroviruses may be shed into the stool for prolonged time after clearance of acute infection, thus limiting the significance of such a finding. RT-PCR methods are now widely available for the detection of viral genomes. However, sequence variation among the different enterovirus groups can lower the specificity and PCR-based assays should regularly be updated using latest sequence information. Serological methods for the detection of enterovirus-specific antibodies are exclusively available in reference laboratories and cannot be used for rapid diagnosis.

#### Parechovirus

Parechoviruses have gained recent interest, but their role in acute gastroenteritis and persistent diarrhoea has yet to be established [173,174]. At the time of writing, 16 parechoviruses types have been described. They now represent an own genus within the familiy *picornaviridae* and real-time RT-PCR is the method of choice for diagnosis in high-income settings [175].

#### Human rotavirus

Rotavirus infection alone is believed to account for 453,000 deaths annually in children younger than 5 years [5]. In most cases, infection causes acute diarrhoea and vomiting with viral particles being shed in high concentrations. Virus isolation is possible on MA104 or CaCO-2-cells but remains laborious and time-consuming. Antigen detection by EIA methods is the current standard procedure for the rapid diagnosis of rotavirus infection and widely available for diagnosis as well as surveillance. These assays are able to detect virus particles even if their concentration is below 10^4^ particles/ml stool suspension. Molecular methods are also available [176,177].

#### HIV-associated enteropathy

HIV-associated enteropathy frequently occurs in HIV-infected individuals without access to antiretroviral therapy and is characterised by persistent diarrhoea, weight loss, anorexia, abdominal pain and dysphagia. HIV-associated enteropathy should be diagnosed by obtaining intestinal biopsies via endoscopy with subsequent histological and microbiological examination [178]. Antiretroviral treatment of the HIV infection usually also cures the enteropathy.

## Discussion

Persistent digestive disorders are unspecific clinical complaints which are commonly reported by many patients around the world. Gastrointestinal or systemic infections are important causes of such disorders with a broad spectrum of possible pathogens involved, including bacteria, intestinal protozoa, helminths and viruses. Due to the wide range of infectious agents which are often difficult to diagnose, great efforts have to be made to reach satisfactory detection rates and to avoid overlooking of important pathogens. Such a diagnostic work-up should include bacterial stool cultures on different selective media (including MacConkey, sorbitol-MacConkey, Leifson and other agar plate cultures), microscopic examination of unstained (e.g. direct faecal smear, Kato-Katz thick smear and formalin ether-concentration method) and stained microscope slides (acid-fast stains, e.g. Kinyoun technique) for parasite identification, and various pathogen-specific tests such as PCR for viruses and diarrhoeagenic *E. coli* pathotypes, toxin detection kits for *C. difficile* diagnosis, and stool concentration methods for *S. stercoralis* (e.g. Baermann funnel and Koga agar plate). Examination of more than one stool specimen over consecutive days is crucial, because many intestinal pathogens are irregularly shed in the faeces [149]. ‘Classical’ approaches to persistent diarrhoea often lead to disappointing results with up to 80% of cases in which no causative pathogen can be determined [6].

However, even exhaustive laboratory work-up is prone to a host of limitations and challenges that must be considered and addressed. Firstly, gastrointestinal complaints are often caused by non-infectious causes, and a combination of different clinical signs and symptoms as well as further tests are needed to detect and exclude such non-infectious aetiologies. Secondly, available epidemiological data regarding the sought infectious pathogens in the tropics are scarce, thus requiring broad diagnostic testing to avoid overlooking of important pathogens. Thirdly, studies should be carried out in different social-ecological settings to assess the influence of cultural, demographic, genetic, geographic, socioeconomic and health system related factors on predominating pathogens. Fourthly, such research must address all pathogen classes and should not be limited to one-dimensional approaches examining either bacteria or parasites only. Fifthly, there are certain issues unique to gastrointestinal diseases which clearly distinguish them from other organ disorders; most importantly, the finding of a given pathogen may not necessarily mean that the patient’s complaints are caused by this organism [179]. Bacteria, helminths and intestinal protozoa may often be found as harmless commensals or even beneficial parts of the gastrointestinal flora, and thus such findings may represent coincidence rather than causality [180-182]. This is of particular importance when different potential pathogens are found concurrently in one faecal specimen and the causative one(s) have to be differentiated. Sixthly, even primarily non-intestinal infectious pathogens may cause gastrointestinal symptoms, as has been reported for HIV infection and even malaria in the tropics, where acute or long-lasting diarrhoea may be the only symptom in up to 20% of all observed cases [183,184]. In contrast, patients may as well start to complain about reduced well-being and develop clinical symptoms only some weeks to months after clearance of an intestinal pathogen, as is the case in postinfectious irritable bowel syndrome [185]. Finally, the variety of possible pathogens affecting the gut is so exhaustive that even very sophisticated diagnostic approaches will not be able to detect every pathogen with satisfactory sensitivity and specificity, especially when considering the cost and practical applicability of some specialised techniques that are not currently feasible in most parts of the tropics.

## Conclusion

There is a pressing need for research targeting persistent digestive disorders as a coherent clinical problem rather than as a disconnected collection of pathologies. This would allow the elaboration of evidence-based diagnosis-treatment algorithms centred on patients in resource-constrained settings, where data availability is scarce and patient management often driven by experience and local beliefs. This is the overarching goal of the NIDIAG consortium, focusing on digestive disorders as discussed here, as well as on neurological disorders [23] and persistent fever [24]. Additionally, such investigations will optimise the use of existing diagnostic tests and advance the development of new methods, which are ideally able to concurrently detect a broad spectrum of intestinal pathogens with a high sensitivity and specificity, and which are simple and affordable enough to be performed in low-income countries where prevalences of persistent digestive disorders are generally high. Moreover, the thorough evaluation of reference tests for intestinal pathogens can serve as diagnostic ‘gold’ standard in the standardisation and validation of easily applicable RDTs, which are highly needed tools in resource-constrained field settings. Finally, such in-depth investigations are not only important for individual patient management, but also for public health policy making (e.g. to assess the efficacy and cost-effectiveness of ongoing preventive chemotherapy control programmes targeting helminthiases). There is a need for improved diagnostics for persistent digestive disorders in the tropics. It is desirable to conduct a multicentric study to investigate the clinical presentations and respective identified pathogens of large patient cohorts presenting with non-acute gastrointestinal diseases as a first step towards more reliable and evidence-based clinical case management in the tropics.

## Competing interest

The authors declare that they have no competing interests.

## Authors’ contributions

SLB, JV and JU took primary responsibility for the literature search. SLB, JV, SK, MP and JU drafted the manuscript. According to their areas of expertise, the authors critically revised the text chapters (bacteria: SLB, DCW, LvM, CPY and MAM; parasites: SLB, SK, DCW, KP, HM, MS, FM, MAM, LvL, EKN and JU; viruses: MP; clinical aspects: SLB, CPY, JJ, EB and SR). All authors contributed to the manuscript, read and approved the final version.

## Financial support

This work is part of the NIDIAG network (Collaborative Project; http://www.nidiag.org) supported by the European Commission under the Health Cooperation Work Programme of the 7th Framework Programme (grant agreement no. 260260).

## Pre-publication history

The pre-publication history for this paper can be accessed here:

http://www.biomedcentral.com/1471-2334/13/37/prepub
